# Cellular Alterations in Immune Checkpoint Inhibitor Therapy-Related Cardiac Dysfunction

**DOI:** 10.1007/s11897-024-00652-2

**Published:** 2024-03-02

**Authors:** Lars Michel, Peter Ferdinandy, Tienush Rassaf

**Affiliations:** 1 Department of Cardiology and Vascular Medicine, West German Heart and Vascular Center, University Hospital Essen, Hufelandstr. 55, 45147 Essen, Germany; 2 Department of Pharmacology and Pharmacotherapy, Semmelweis University, Budapest, Hungary; 3 Pharmahungary Group, Szeged, Hungary

**Keywords:** Cardio-oncology, Cardiotoxicity, Hidden cardiotoxicity, Immune checkpoint inhibitor, Immune-related adverse events, Programmed cell death protein 1

## Abstract

**Purpose of Review:**

Immune checkpoint inhibitor (ICI) therapy has emerged as a pivotal advancement in cancer treatment, but the widespread adoption has given rise to a growing number of reports detailing significant cardiovascular toxicity. This review concentrates on elucidating the mechanisms behind ICI-related cardiovascular complications, emphasizing preclinical and mechanistic data.

**Recent Findings:**

Accumulating evidence indicates a more significant role of immune checkpoints in maintaining cardiac integrity than previously understood, and new key scientific data are available to improve our understanding of ICI-related cardiovascular toxicity, including hidden cardiotoxicity. New avenues for innovative concepts are hypothesized, and opportunities to leverage the knowledge from ICI-therapy for pioneering approaches in related scientific domains can be derived from the latest scientific projects.

**Summary:**

Cardiotoxicity from ICI therapy is a paramount challenge for cardio-oncology. Understanding the underlying effects builds the foundation for tailored cardioprotective approaches in the growing collective at risk for severe cardiovascular complications.

## Introduction

Immune checkpoints are signaling pathways that exert an inhibitory function on the immune system. They lead to an inhibition of T-cell activity by reducing proliferation and triggering apoptosis. The physiological function of immune checkpoints is to prevent excessive immune reactions and autoimmune reactions. In many cancers, however, activated immune checkpoint signaling pathways lead to an inhibition of anticancer immune response. The mechanism of cancer cells to prevent an antitumor immune response is considered one of the key features of carcinogenesis and has defined as a hallmark of cancer. Immune checkpoint inhibitor (ICI) therapy aims to restore an antitumor immune response by blocking tumor-related immune checkpoint signaling thus preventing immune evasion [1, 2].

The programmed cell death protein 1 (PD-1) gene was first identified in 1992 by Tasuko Honjo in two hematopoietic cell lines after studying effectors of the classical type of programmed cell death [3]. PD-1 was later shown to be activated mainly by its ligand PD-L1 and to specifically inhibit T-cell immunologic response as a so-called immune checkpoint which together play a key role in regulating the activity of the immune system [4, 5]. PD-1 is located on subtypes of T and B cells, whereas PD-L1 has been described extensively in the kidneys, heart, spleen, and to a lesser extent in various peripheral tissues. PD-1 triggers inhibitory signals in most cases, which inhibit the activity of the immune system. It primarily influences intercellular interactions of various immune cells and promotes the differentiation of immunosuppressive Tregs [6]. The preclinical findings eventually led to the development of the concept of immune ICI therapy [2, 5].

Cytotoxic T-lymphocyte-associated protein 4 (CTLA-4) is a receptor on T cells that regulates immune responses. Its main functions include inhibiting T-cell activation by competing with CD28 for binding to CD80/CD86 on antigen-presenting cells. Thereby, CD28-mediated co-stimulation of T-cell activation is prevented, which leads to a downregulating of the immune response, induction of peripheral tolerance to prevention of autoimmune reactions [7]. The first evidence of an enhancement of antitumor immunity was published in 1996 by the group of James P. Allison which later led to the development of anti-CTLA-4 ICI therapy [8].

ICI therapy represents the most important milestone in oncology over the past decade because of its global use and rapidly growing patient numbers. It is considered the most innovative form of cancer treatment of treatment [7]. As a result of continuous scientific development and the expansion of clinical application, their use is steadily increasing. New substances are continuously developed, and the treatment indication is expanded to new types of cancer (Table 1) [9]. The quantitative analysis of patients with cancer and a potential indication for ICI therapy according to approval clearly shows the scope of the development: The estimated percentage of patients who were eligible for ICI therapy increased from 1.54% in 2011 to 43.63% in 2018 [10]. At the same time, the growing use of immunotherapies has led to an increased awareness of side effects, the so-called immune-related adverse events (irAEs) [11, 12].

**Table 1:** Immune checkpoint inhibitor drugs on the market

Target structure	Drug (generic name and marketed brand name)
Anti-CTLA-4	Ipilimumab (Yervoy®): EMA and FDA approval: 2011 Tremelimumab (Imjudo®) EMA approval: 2023 FDA approval: 2022
Anti-PD-1	Nivolumab (Opdivo®) EMA approval: 2015 FDA approval: 2014 Pembrolizumab (Keytruda®) EMA approval: 2015 FDA approval: 2014
Anti-PD-L1	Avelumab (Bavencio®) EMA and FDA approval: 2017 Atezolizumab (Tecentriq®) EMA approval: 2017 FDA approval: 2016 Durvalumab (Imfinzi®) EMA approval: 2018 FDA approval: 2017
Anti-LAG-3	Relatlimab (Relatlimab + Nivolumab: Opdualag®) EMA and FDA approval: 2022

*CTLA-4* cytotoxic T-lymphocyte-associated protein 4, *EMA* European Medicines Agency, *FDA* Food and Drug Administration, *LAG-3* lymphocyte activation gene 3, *PD-1* programmed cell death protein 1, *PD-L1* programmed cell death protein 1 ligand 1

All organ systems can be affected by irAEs that are triggered by exaggerated immune reactions and autoimmune reaction. They range from mild symptoms to life-threatening conditions. Most commonly, irAEs involve the skin, the gut, the lung, and the liver. They are graded by the Common Terminology Criteria of Adverse Events [13]. While cardiovascular adverse events were initially considered rare, they show the highest fatality rates compared to any other organ manifestation [11].

## Cardiovascular Immune-Related Adverse Events

**Fig. 1:**
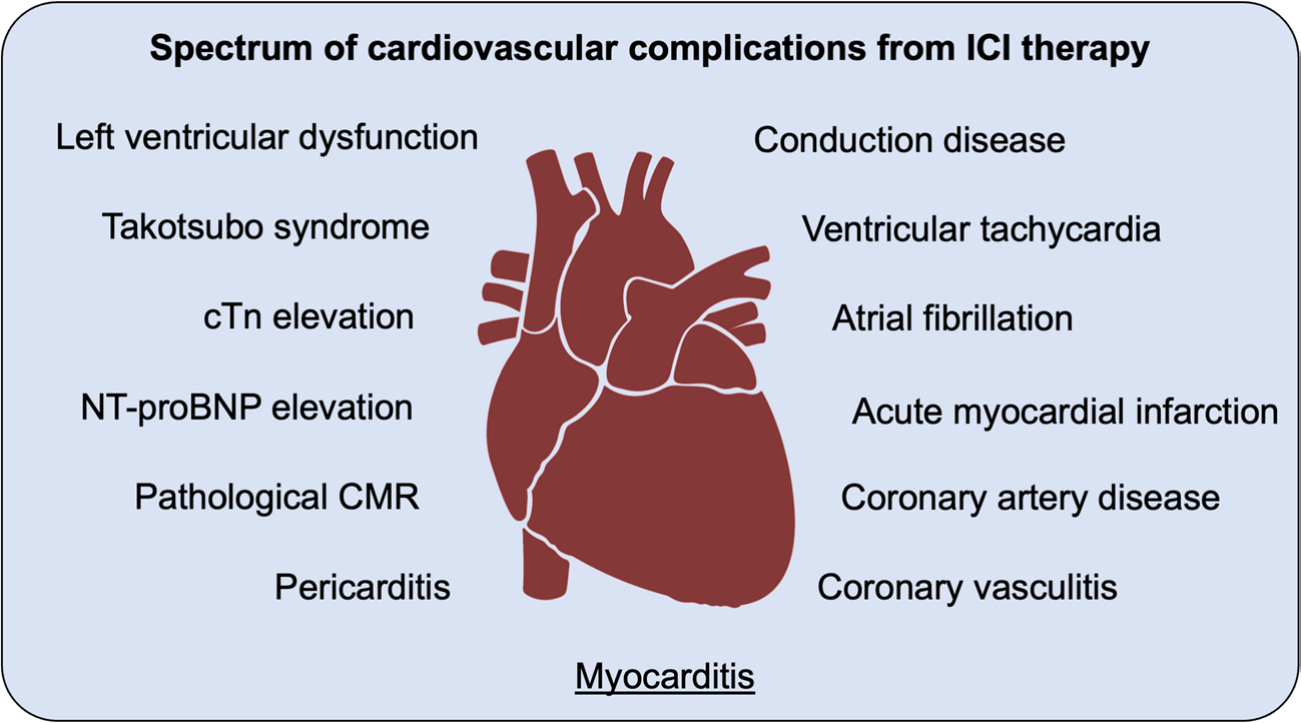
Spectrum of cardiovascular complications from ICI therapy. CMR, cardiac magnetic resonance imaging; cTn, cardiac troponin; NT-proBNP, *N*-terminal pro-brain natriuretic peptide; ICI, immune checkpoint inhibitor

Cardiovascular adverse events from ICI therapy represent a major issue in ICI therapy and have emerged as one of the main challenges in cardio-oncology. First cases of cardiotoxicity were reported in 2016 [14, 15]. Early reports showed cases of severe myocarditis with high lethality but negligible incidence. Throughout the next years, the increasing application of ICI therapy and the raised awareness on potential cardiovascular side effects led to increasing reports on myocarditis with a reported incidence of 1.14% in 2018 [16]. Further analyses showed that ICI-related myocarditis can show a heterogeneous presentation with moderate to fulminant signs and symptoms. Additionally, other forms of cardiotoxicity other than myocarditis were described during ICI therapy including left ventricular dysfunction, pericarditis, takotsubo syndrome, and isolated arrhythmia [17]. Additionally, an association to accelerated atherosclerosis with a subsequently increased risk for coronary artery disease and acute coronary syndromes was demonstrated [18]. Systematic registries have revealed a much higher incidence than previously expected. The on-year incidence of cardiac events in patients receiving ICI therapy ranged from 6.6 to 9.7% in a Danish registry-based analysis [19]. Prospective data with large collectives are still missing which poses an uncertainty about causal effects or mere associations, independent occurrence through common risk factors, or previously unknown third factors favoring the occurrence of cardiovascular events (Fig. 1).

While proinflammatory effects are widely considered to be the main factor leading to cardiovascular events with ICI therapy, the precise underlying pathomechanisms are still being elucidated. There is a high medical need of a better understanding of those mechanisms, eventually aiming to provide evidence that supports improved risk assessment, diagnosis, and tailored cardioprotective approaches in the growing collective of patients at risk [20]. Answering the following questions is at the center of the current scientific debate on the mechanisms of ICI-related cardiotoxicity:

1. What is the key factor(s) including comorbidities and comedications that determine why some patients experience cardiovascular side effects and others do not?
2. Are there one or more specific cardiovascular autoantigens that serve as the target in ICI-related cardiovascular toxicity?
3. How do cardiovascular proinflammatory changes lead to manifest organ dysfunction including left ventricular dysfunction, arrhythmia, and atherosclerosis?
4. Can specific pathogenetic components be targeted to prevent or reduce cardiovascular side effects while preventing the antitumor efficacy of ICI therapy?

## Immune Checkpoints in the Heart

It is suspected that cardiac expression of PD-L1 shields the myocardium from inflammatory injury [21]. As a proinflammatory cytokine, interferon gamma (IFN-γ) appears to be the main stimulus for PD-L1 expression on cardiac endothelial cells in an autocrine and paracrine manner. This mechanism, by which an inflammatory signal triggers PD-L1 expression, may explain how heart tissue is protected from T-cell damage during inflammatory diseases [22].

The first evidence for cardiotoxicity associated with immune checkpoints was found in knockout mouse models [23, 24]. PD-1-deficient BALB/C mice exhibit a dilated cardiomyopathy phenotype with fibrous remodeling of the myocardium and severely reduced left ventricular function [24]. They die at the age of 20 weeks. Myocardial and circulating anti-troponin I autoantibodies were identified as the putative underlying pathomechanism [25]. Interestingly, manifest cardiotoxicity appears to be a strain-specific effect: PD1-deficient mice with a C57BL/6 background show normal heart dimensions and a preserved left ventricular function. It is speculated that the globally higher level of immune reactivity in BALB/C mice compared to other strains promotes manifest cardiotoxicity in PD1-deficient mice.

A role for PD-1/PDL-1 in protecting myocardial integrity has furthermore been evaluated in different experimental settings. A proinflammatory effect on the myocardium was shown in autoimmune myocarditis mouse models and in graft arterial disease in cardiac allografts. Here, PD1-deficient T cells exhibited enhanced proliferation and cytotoxic activity resulting in increased myocardial damage [26–28]. In another recent study, PD-1 blockade induced cardiac dysfunction in mice with increased IL-17 signaling in the thymus, and pharmacological inhibition of IL-17A treatment prevented ICI-induced cardiac dysfunction [29]. The PD-1/PDL-1 axis has also been linked to a cardiac parasitic infection. Chagas disease is transmitted by *Trypanosoma cruzi* and can lead to Chagas cardiomyopathy after chronic persistence of the parasites [30]. Inhibition of PD-1 and PD-L1 was found to aggravate myocardial inflammation in chronic Chagas disease in mice after infection with irradiated *Trypanosoma cruzi* indicating that PD-1/PDL-1 interaction may prevent cardiac injury by excessive inflammation driven by infiltrating T cells [30]. Upregulation of PD-L1 on cardiac endothelium was observed in response to acute lymphocytic myocarditis, and PD-L1 depletion aggravated the disease phenotype further indicating a role of PD-1/PD-L1 signaling in controlling immune-mediated cardiac injury [22]. In addition, there is first evidence that PD-L1 is upregulated in human myocardium during idiopathic lymphocytic myocarditis but no systematic data is yet available [21].

CTLA-4-deficient mice show a similar, yet more pronounced phenotype. CTLA-4-deficient mice develop multi-organ lymphocytic infiltration and die at 3–4 weeks of age [23, 31]. In contrast to PD-1^−/−^ mice that only show myocardial immunoglobulin G (IgG) deposits, CTLA-4^−/−^ mice display manifest, severe infiltration of activated (CD44^+^ interleukin (IL)-2Rα^+^) lymphocytes within the myocardium and signs for acute cardiomyocyte necrosis. Interestingly, manifest cardiotoxicity appears to occur in CTLA-4^−/−^ mice with both C57BL/6 and BALB/C background, but the severity has not yet been systematically studied [23]. Autoimmune reactivity in CTLA-4-deficient mice shows distinct organ tropism in different forms of CTLA-4 depletion. Particularly, T cells from CTLA-4^−/−^ mice that were transferred to wild-type littermates infiltrated the myocardium of recipients but CTLA-4-depletion by *Cre recombinase* in a conditional CTLA-4 knockout mouse model did not induce myocardial lymphocyte infiltration [32]. Cardiac implications of CTLA-4 expression in humans have not been extensively studied. In a small single-center study, a CTLA-4 single-nucleotide polymorphism was found to be associated with the development of dilated cardiomyopathy, but the underlying mechanisms are not yet understood [33]. In fact, immune checkpoints are promising molecular drug candidates to treat heart failure as forwarded in various preclinical models by modulating inflammation and myocardial fibrosis [34, 35]. Therefore, it may be challenging to keep the anticancer effect of ICI inhibitors without potential cardiotoxic effects.

## Cardiac Involvement During ICI Therapy

Experimental and clinical observations have addressed the question of why adverse effects on the cardiovascular system have the highest mortality of all types of organ complications. One possible explanation lies in the large contact surface of the myocardium with the immune system. The myocardium is highly capillarized—each cardiomyocyte is in contact with an average of six capillaries. This creates a particularly large contact surface, which can make the heart vulnerable to even minor inflammatory changes, e.g., the diapedesis of ICI-dependent activated T cells from the bloodstream into the myocardium [21].

Multiple experimental models have shown cardiac involvement upon dysregulation of immune checkpoint signaling in otherwise healthy specimen and cardiovascular disease. It is therefore prompting to speculate that ICI therapy induces a general cardiac phenotype which leads to overt cardiotoxicity in the presence of further predisposing factors, and this association was recently assessed in a newly designed preclinical model [36, 37].

The unique feature was the generation of a melanoma mouse model which showed response to anti-PD-1 ICI therapy. Presence of cancer can substantially alter the cardiocirculatory and immunological function by inducing a catabolic state, proinflammatory changes, and hemodynamic demands [38]. Hence, assessing cardiotoxic effects in tumor models will provide higher validity compared to the use of models with otherwise healthy specimen. Mice were transplanted with a melanoma cell line followed by anti-PD-1 therapy for two weeks. In the melanoma model, functional analysis using echocardiography and pressure volume catheterization showed a small yet significant decrease in systolic function which was augmented after inotropic stress with dobutamine. A similar effect was recapitulated in patients during the early phase of ICI therapy including a PD-1 inhibitor showing a decrease in systolic function compared to baseline. Interestingly, mice without tumor that received anti-PD-1 ICI therapy did not show a deterioration of left ventricular function [36•].

The analysis of cardiac immune cells revealed that treated mice show increasing numbers of activated CD4^+^ and CD8^+^ T cells in the myocardium compared to controls. Aiming to understand how immune cell infiltration led to changes in cardiac function, a holistic mass spectrometry–based approach was applied, incorporating the simultaneous analysis of proteomics, metabolomics, and lipidomics [39]. The analysis showed profound changes in different metabolic pathways, most of them related to energy metabolism. The lipid metabolism was altered in the form of an accumulation of beta oxidation substrates together with a reduced carnitine/acylcarnitine shuttle, acyl-CoA dehydrogenase, and acyl-CoA synthetase, indicating a reduced use of lipids in myocardial energy metabolism. Glucose metabolism was also altered particularly regarding pyruvate transport and glycolysis. On the level of mitochondria, changes in enzymes critical for oxidative phosphorylation were observed including decreased NADH dehydrogenase (complex I of the respiratory chain), decreased ATP synthase (complex V of the respiratory chain), and decreased FAD synthase. Phosphocreatine was elevated as a quick mobilizable energy supplier in striated muscles.

In another project, an interesting observation was achieved regarding cardiac macrophages following in vivo anti-PD-1 ICI therapy [40]. Following therapy, the number of M1-polarized macrophages indicated by staining for the inducible nitric oxide synthase (iNOS) was markedly increased compared to controls as a sign for increased inflammatory activity. Further analyses determined that miR-34a was more abundant in cardiac tissue following anti-PD-1 ICI treatment. Experimental miR-34a inhibition led to decreased numbers of iNOS-positive M1 macrophages and even prevented an impairment of left ventricular function. However, these experiments were conducted in a tumor-free model, hence limiting transfer to the situation in cancer patients [40].

Radiotherapy and ICI therapy are routinely applied in common cancers, including lung cancer. Whether radiotherapy involving parts of the heart that induces subclinical myocardial injury that aggravates the inflammatory effects of ICI-therapy was assessed in a recent experimental project [41]. Here, mice were treated with anti-PD-1 ICI therapy while receiving chest radiation involving the heart. The number of infiltrating CD4 + and CD4 + T cells was increased with a more severe reduction in left ventricular function. Ultrastructural analysis showed increased fibrosis in the myocardium [41].

Evidence on human myocardium is sparse and predominantly limited to case reports and small case series [15, 42]. One analysis should be emphasized in this context due to its innovative approach: To understand changes that are specific to ICI-related myocarditis, the authors performed RNA sequencing in endomyocardial biopsy samples from patients with ICI-related myocarditis compared to those with common viral myocarditis and dilated cardiomyopathy [43]. Interferon-γ-dependent pathways were identified to significantly altered in ICI-related myocarditis samples with a specific marked increase of guanylate binding protein (GBP)-5 and 6. GBP-5 is a strong inductor NLRP3 inflammasome activity which plays a key role in inflammatory signaling cascades including antigen detection and IL-dependent induction of inflammation [44]. The clinical data is supported by preclinical observations showing increased IL-1α, IL-4, and phosphorylated extracellular signal-regulated kinases (ERK) as a sign for globally upregulated proinflammatory cytokines upon immune checkpoint inhibition/depletion in the heart [45].

## The Two-Hit Hypothesis—Hidden Cardiotoxicity

Based on multiple preclinical models, it becomes evident that the manifestation of cardiotoxicity from ICI therapy is dependent on further predisposing factors. Those factors could be an underlying cardiac disease that aggravates from ICI therapy (myocarditis mouse mode, Chagas cardiomyopathy), the induction of subclinical cardiac injury (chest radiation mouse model), systemic stressors in the form of a growing tumor (melanoma mouse model), or an immune-sensitive condition (the BALB/C mouse strain). This association may also account for the fact that preclinical studies on ICI therapy before the large clinical application did not demonstrate major adverse effects in vivo [7]. Paralleling preclinical evidence, the analysis of cardiovascular irAEs in large collective shows great heterogeneity with no overt cardiovascular phenotype in most patients, while a fraction develops fulminant complications with high mortality. Until now, classical cardiovascular risk factors have not convincingly shown an association with cardiovascular complications. The use of dual ICI therapy (PD-1 inhibitor plus CTLA-4 inhibitor) has remained the only reliable risk factor for cardiovascular toxicity.

It is tempting to speculate that ICI therapy induces latent changes on a molecular and immunological level that progress to manifest cardiovascular toxicity in the presence of a further predisposing condition or risk factors as a *Second Hit*. Any form of cardiac stress may render the heart vulnerable to the proinflammatory effects of ICI therapy by reducing the ability to compensate for stressors or by exposing cardiovascular structures as epitopes to the immune system that may initiate autoimmune phenomena. Indeed, this phenomenon has recently been termed “hidden drug cardiotoxicity.” The concept is that the cardiotoxicity of drugs with cardiotoxic potential only manifests in the diseased heart with pre-existing structural damage due to pre-existing cardiac disease with cellular dysfunction, metabolic changes, and alterations in signaling pathways associated with its major comorbidities. It is proposed that these effects reduce endogenous cardioprotection, promoting the manifestation of drug-induced adverse effects. Notably, this concept may explain why cardiotoxicity has been underreported in pre-approval studies due to study collectives with a low incidence of pre-existing cardiovascular disease [46, 47

## Tumor Necrosis Factor Alpha

Depletion of the PD-1/PD-L1 pathway either by genetic depletion or antibody therapy is commonly associated with increased levels of a variety of proinflammatory cytokines. Various analyses highlight an exposed role of tumor necrosis factor alpha (TNFα) as a master switch in mediating cardiotoxicity. TNFα is upregulated in cardiac tissue and in serum of mice receiving anti-PD-1 ICI therapy. Following radiation injury, TNFα expression is further augmented in the heart and as the membrane-bound isoform in T cells. TNFα is a potent stimulus to induce upregulation of PD-L1 but leads to a decrease of the secondary immune checkpoint T-cell immunoglobulin and mucin-domain containing (TIM-3) in cancer tissue [48, 49]. Cardiomyocyte expression of TNFα is predicted to mediate cardiac function, with high TNFα expression leading to negative inotropic effects in vitro and in vivo. As putative mechanisms, sympathetic β-adrenoceptor activation and negative effects on the L/T-type calcium channel expression and activity have been postulated. Heart failure patients commonly show increased TNFα levels further linking inflammation from PD-1 blockade and cardiac functional capacity [50].

Immunosuppressive treatment is effective in treating irAEs from ICI therapy but reduces or neutralizes the anticancer efficacy. It is therefore particularly problematic when evaluating prophylactic approaches that prevent the progression from subclinical molecular changes to manifest complications. Anti-TNFα therapy may serve as an innovative new treatment that serves to modulate inflammation while at the same time interfering with immune checkpoint expression. TNFα blockade was already shown to aid anti-PD-1 therapy in experimental melanoma by augmenting response to therapy and preventing autoimmune colitis in mice [36, 48, 49

## The Antigen in ICI-Related Cardiotoxicity

Deciphering the target antigen represents a pivotal pursuit within the realm research on ICI-related cardiotoxicity. While autoantibodies against cardiac troponin I were identified in PD-1 knockout mice, no such antibodies are routinely found in patients [25]. The best available evidence to date is based on a genetic mouse model for myocarditis by inducing a heterozygous genetic depletion of CTLA-4 together with a homozygous depletion of PD-1 [51•]. Genetically modified mice display a phenotype which is comparable to ICI-related myocarditis. A special feature of this model is the heterogeneous course: some test animals develop only a moderate course, while others show a fulminant manifestation. Additionally, this is the first mouse model which shows concomitant arrhythmia (e.g., atrioventricular block) which is common in patients but was never resembled in a preclinical model before [51•]. However, the absence of a tumor can be seen as a significant weakness.

By applying a very elegant approach, candidate genes were identified and further analyzed, eventually leading to the identification of ɑ-myosin as the target of CD8^+^ T cells. In a translational approach, peripheral blood T cells from three patients with ICI-related myocarditis showed a strong induction of expansion by ɑ-myosin peptides. While first evidence is very convincing, analyses in bigger cohorts are warranted to recapitulate the finding and to assess further putative antigens [51, 52].

**Fig. 2:**
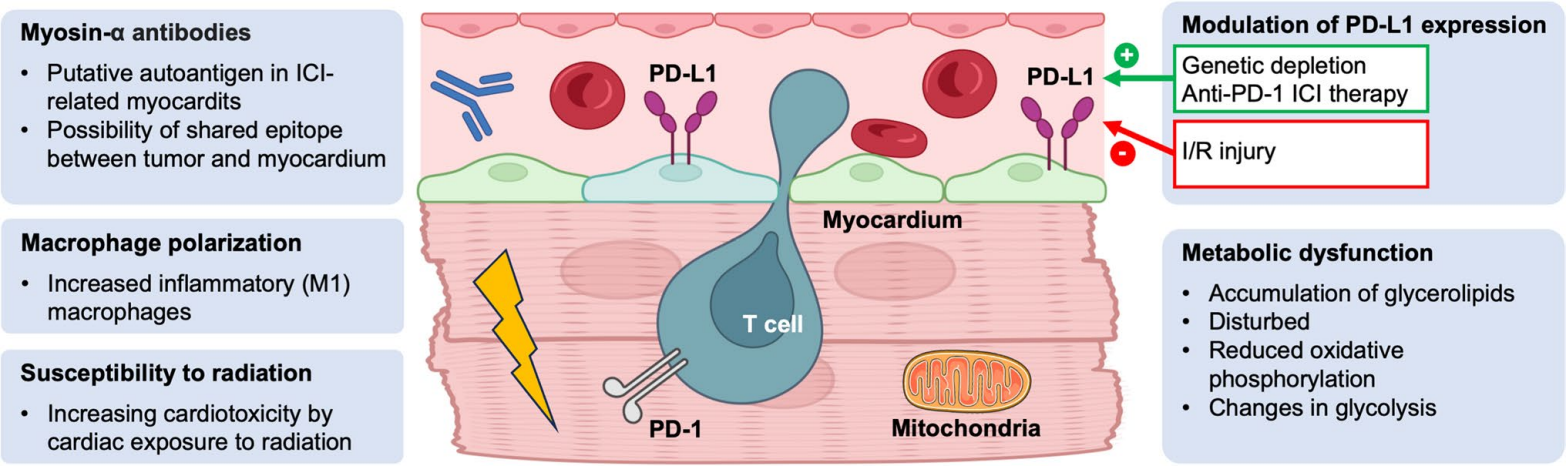
Mechanisms of cardiotoxicity associated with PD-1. ICI, immune checkpoint inhibitor; I/R injury, ischemia/reperfusion injury; PD-1, programmed cell death protein 1; PDL1, programmed cell death protein 1 ligand 1

Another clue to potential antigens comes from one of the first publications on ICI-related myocarditis in 2016, which described two cases with fulminant, ultimately fatal disease [15]. In one patient, next-generation sequencing of the T-cell receptor in cardiac, skeletal muscle, and tumor tissue revealed that the same most abundant T-cell receptor was present in both the tumor and cardiac tissue. Whole transcriptome sequencing revealed the expression of muscle-specific transcripts in tumor tissue. It is therefore tempting to speculate whether a shared epitope in the tumor and cardiac tissue or some form of antigenic mimicry plays a role in the pathogenesis of ICI myocarditis. Strikingly, the relevant T-cell receptor clone was already present in the tumor pre-treatment, raising the question of whether the analysis of cardiac-specific T-cell clones in tumor biopsy samples pre-treatment could be used to identify patients at high risk of ICI-related myocarditis. However, these findings have not been replicated, and such speculation is highly speculative based on the available evidence (Fig. 2) [15].

## Coronary Artery Disease and Acute Myocardial Infarction

Growing evidence from both preclinical and clinical research suggests a link between ICI therapy and the onset of atherosclerosis. The suspected influence on chronic inflammation is believed to be a pivotal factor in the formation of atherosclerotic plaques [18, 53]. In a mouse model designed to accelerate atherosclerosis through genetic depletion of the low-density lipoprotein (LDL) receptor, it was demonstrated that the use of anti-CTLA-4 and anti-PD-1 ICI therapy increased the size of atherosclerotic plaques and altered their composition. This treatment resulted in an elevated presence of CD8^+^ T cells within the plaques with subsequently increased sizes of the necrotic core, indicating heightened chronic inflammation. The infiltration of T cells [54] was likely triggered by increased integrin expression (vascular cell adhesion molecule 1 and intercellular adhesion molecule 1). The authors infer that this mechanism accelerates atherosclerosis, potentially contributing to the onset and exacerbation of coronary heart disease. The heightened inflammation in the plaques could also enhance instability, thereby elevating the risk of plaque rupture and the development of acute coronary syndrome [12, 18]. Concurrently, retrospective analyses of patients undergoing ICI therapy reveal a significant impact. A paired cohort study demonstrated a threefold increase in the risk of cardiovascular events after initiating ICI therapy. In a sub-study, the progression of aortic plaque size was found to be three times higher under ICI therapy. Prospective studies will be essential in determining the reproducibility of such associations [55, 56].

Myocardial ischemia/reperfusion (I/R) injury following acute myocardial infarction involves a sterile myocardial inflammation that is primarily governed by myeloid cells followed by infiltrating lymphocytes [57]. Despite a potential role of immune checkpoints in this process, only limited knowledge is available on the role of PD1/PDL1 in I/R injury. In a recent study examining PD1 and PDL1 in an ex vivo rat model of I/R injury and cryo-injury using in Langendorff-perfused hearts, increased expression of PDL1 on cardiac cells was found in response to myocardial injury [58]. However, this finding is limited as it has been performed ex vivo in absence of a humoral immune system, and the variety of different cardiac cell subpopulations including endothelial cells and fibroblasts was not considered. Data on the role of PD-1/PD-L1 in an in vivo I/R injury mouse model is available from one project. Flow cytometry and immunofluorescence data confirmed that cardiac PD-L1 is mainly expressed on cardiac endothelial cells rather than cardiomyocytes [22, 45]. Upon cardiac I/R-injury, endothelial PD-L1 expression gradually decreases during the first 3 days of reperfusion, indicating a relevant role in cardiac injury and repair. In immunofluorescence, decreasing PD-L1 can specifically be attributed to the infarcted area. Anti-PD-1 ICI therapy during I/R injury induced an increasing number of infiltrating CD8 + T cells, but the clinical consequences not understood so far [45]. Also, insufficient evidence on the presumably significant role CTLA-4 in this scenario is available. Further research is needed to determine the significance of the PD-1/PD-L1 immune checkpoint pathway in acute myocardial infarction, to assess the clinical relevance, and to evaluate a potential therapeutic or prognostic relevance [45].

## Conclusion

ICI therapy has evolved into a cornerstone in the treatment of diverse cancers, emerging as a pivotal and revolutionary modality in cancer therapy. Consequently, addressing and effectively managing potentially life-threatening cardiovascular complications have become a paramount challenge in contemporary cardio-oncology. The exploration of mechanisms underpinning ICI-related cardiovascular complications not only holds critical importance in enhancing patient safety in oncology but also presents an opportunity to leverage this knowledge for pioneering approaches in related scientific domains, such as acute myocardial infarction. Advancing our comprehension of the role of immune checkpoints in cardiovascular disease extends the boundaries of our pathophysiological understanding, promising to refine and elevate the treatment strategies available to patients.

## Data Availability

No datasets were generated or analyzed during the current study.

## References

[1] Pardoll DM (2012). The blockade of immune checkpoints in cancer immunotherapy. Nat Rev Cancer.

[2] Sharma P, Allison JP (2020). Dissecting the mechanisms of immune checkpoint therapy. Nat Rev Immunol.

[3] Ishida Y, Agata Y, Shibahara K, Honjo T (1992). Induced expression of PD-1, a novel member of the immunoglobulin gene superfamily, upon programmed cell death. Embo J.

[4] Freeman GJ, Long AJ, Iwai Y, Bourque K, Chernova T, Nishimura H, Fitz LJ, Malenkovich N, Okazaki T, Byrne MC, Horton HF, Fouser L, Carter L, Ling V, Bowman MR, Carreno BM, Collins M, Wood CR, Honjo T (2000). Engagement of the PD-1 immunoinhibitory receptor by a novel B7 family member leads to negative regulation of lymphocyte activation. J Exp Med.

[5] Gong J, Chehrazi-Raffle A, Reddi S, Salgia R (2018). Development of PD-1 and PD-L1 inhibitors as a form of cancer immunotherapy: a comprehensive review of registration trials and future considerations. J Immunother Cancer.

[6] Francisco LM, Sage PT, Sharpe AH (2010). The PD-1 pathway in tolerance and autoimmunity. Immunol Rev.

[7] Waldman AD, Fritz JM, Lenardo MJ (2020). A guide to cancer immunotherapy: from T cell basic science to clinical practice. Nat Rev Immunol.

[8] Leach DR, Krummel MF, Allison JP (1996). Enhancement of antitumor immunity by CTLA-4 blockade. Science.

[9] Maritaz C, Broutin S, Chaput N, Marabelle A, Paci A (2022). Immune checkpoint-targeted antibodies: a room for dose and schedule optimization?. J Hematol Oncol.

[10] Haslam A, Prasad V (2019). Estimation of the percentage of US patients with cancer who are eligible for and respond to checkpoint inhibitor immunotherapy drugs. JAMA Netw Open.

[11] Wang DY, Salem JE, Cohen JV, Chandra S, Menzer C, Ye F, Zhao S, Das S, Beckermann KE, Ha L, Rathmell WK, Ancell KK, Balko JM, Bowman C, Davis EJ, Chism DD, Horn L, Long GV, Carlino MS, Lebrun-Vignes B, Eroglu Z, Hassel JC, Menzies AM, Sosman JA, Sullivan RJ, Moslehi JJ, Johnson DB (2018). Fatal toxic effects associated with immune checkpoint inhibitors: a systematic review and meta-analysis. JAMA Oncol.

[12] Poels K, van Leent MMT, Boutros C, Tissot H, Roy S, Meerwaldt AE, Toner YCA, Reiche ME, Kusters PJH, Malinova T, Huveneers S, Kaufman AE, Mani V, Fayad ZA, de Winther MPJ, Marabelle A, Mulder WJM, Robert C, Seijkens TTP, Lutgens E (2020). Immune checkpoint inhibitor therapy aggravates T cell-driven plaque inflammation in atherosclerosis. JACC CardioOncol.

[13] Insitute NC. Common terminology criteria for adverse events (CTCAE). 2023. https://ctep.cancer.gov/protocoldevelopment/electronic_applications/ctc.htm#ctc_60. Accessed 01 Nov 2023.

[14] Heinzerling L, Ott PA, Hodi FS, Husain AN, Tajmir-Riahi A, Tawbi H, Pauschinger M, Gajewski TF, Lipson EJ, Luke JJ (2016). Cardiotoxicity associated with CTLA4 and PD1 blocking immunotherapy. J Immunother Cancer.

[15] Johnson DB, Balko JM, Compton ML, Chalkias S, Gorham J, Xu Y, Hicks M, Puzanov I, Alexander MR, Bloomer TL, Becker JR, Slosky DA, Phillips EJ, Pilkinton MA, Craig-Owens L, Kola N, Plautz G, Reshef DS, Deutsch JS, Deering RP, Olenchock BA, Lichtman AH, Roden DM, Seidman CE, Koralnik IJ, Seidman JG, Hoffman RD, Taube JM, Diaz LA, Anders RA, Sosman JA, Moslehi JJ (2016). Fulminant myocarditis with combination immune checkpoint blockade. N Engl J Med.

[16] Mahmood SS, Fradley MG, Cohen JV, Nohria A, Reynolds KL, Heinzerling LM, Sullivan RJ, Damrongwatanasuk R, Chen CL, Gupta D, Kirchberger MC, Awadalla M, Hassan MZO, Moslehi JJ, Shah SP, Ganatra S, Thavendiranathan P, Lawrence DP, Groarke JD, Neilan TG (2018). Myocarditis in patients treated with immune checkpoint inhibitors. J Am Coll Cardiol.

[17] Lyon AR, Yousaf N, Battisti NML, Moslehi J, Larkin J (2018). Immune checkpoint inhibitors and cardiovascular toxicity. Lancet Oncol.

[18] Poels K, Neppelenbroek SIM, Kersten MJ, Antoni ML, Lutgens E, Seijkens TTP. Immune checkpoint inhibitor treatment and atherosclerotic cardiovascular disease: an emerging clinical problem. J Immunother Cancer. 2021;9(6):e002916. 10.1136/jitc-2021-002916. 10.1136/jitc-2021-002916 PMC8231062 34168005

[19] D'Souza M, Nielsen D, Svane IM, Iversen K, Rasmussen PV, Madelaire C, Fosbøl E, Køber L, Gustafsson F, Andersson C, Gislason G, Torp-Pedersen C, Schou M (2021). The risk of cardiac events in patients receiving immune checkpoint inhibitors: a nationwide Danish study. Eur Heart J.

[20] Michel L, Totzeck M, Lehmann L, Finke D. Emerging role of immune checkpoint inhibitors and their relevance for the cardiovascular system. Herz. 2020. 10.1007/s00059-020-04954-8 32533218

[21] Grabie N, Lichtman AH, Padera R (2019). T cell checkpoint regulators in the heart. Cardiovasc Res.

[22] Grabie N, Gotsman I, DaCosta R, Pang H, Stavrakis G, Butte MJ, Keir ME, Freeman GJ, Sharpe AH, Lichtman AH (2007). Endothelial programmed death-1 ligand 1 (PD-L1) regulates CD8+ T-cell mediated injury in the heart. Circulation.

[23] Tivol EA, Borriello F, Schweitzer AN, Lynch WP, Bluestone JA, Sharpe AH (1995). Loss of CTLA-4 leads to massive lymphoproliferation and fatal multiorgan tissue destruction, revealing a critical negative regulatory role of CTLA-4. Immunity.

[24] Nishimura H, Okazaki T, Tanaka Y, Nakatani K, Hara M, Matsumori A, Sasayama S, Mizoguchi A, Hiai H, Minato N, Honjo T (2001). Autoimmune dilated cardiomyopathy in PD-1 receptor-deficient mice. Science.

[25] Okazaki T, Tanaka Y, Nishio R, Mitsuiye T, Mizoguchi A, Wang J, Ishida M, Hiai H, Matsumori A, Minato N, Honjo T (2003). Autoantibodies against cardiac troponin I are responsible for dilated cardiomyopathy in PD-1-deficient mice. Nat Med.

[26] Tarrio ML, Grabie N, Bu DX, Sharpe AH, Lichtman AH (2012). PD-1 protects against inflammation and myocyte damage in T cell-mediated myocarditis. J Immunol.

[27] Wang J, Okazaki IM, Yoshida T, Chikuma S, Kato Y, Nakaki F, Hiai H, Honjo T, Okazaki T (2010). PD-1 deficiency results in the development of fatal myocarditis in MRL mice. Int Immunol.

[28] Koga N, Suzuki J, Kosuge H, Haraguchi G, Onai Y, Futamatsu H, Maejima Y, Gotoh R, Saiki H, Tsushima F, Azuma M, Isobe M (2004). Blockade of the interaction between PD-1 and PD-L1 accelerates graft arterial disease in cardiac allografts. Arterioscler Thromb Vasc Biol.

[29] Gergely TG, Kucsera D, Tóth VE, Kovács T, Sayour NV, Drobni ZD, Ruppert M, Petrovich B, Ágg B, Onódi Z, Fekete N, Pállinger É, Buzás EI, Yousif LI, Meijers WC, Radovits T, Merkely B, Ferdinandy P, Varga ZV (2023). Characterization of immune checkpoint inhibitor-induced cardiotoxicity reveals interleukin-17A as a driver of cardiac dysfunction after anti-PD-1 treatment. Br J Pharmacol.

[30] Fonseca R, Salgado RM, da Borges Silva H, do Nascimento RS, D'Império-Lima MR, Alvarez JM (2018). Programmed cell death protein 1-PDL1 interaction prevents heart damage in chronic Trypanosoma cruzi infection. Front Immunol.

[31] Waterhouse P, Penninger JM, Timms E, Wakeham A, Shahinian A, Lee KP, Thompson CB, Griesser H, Mak TW (1995). Lymphoproliferative disorders with early lethality in mice deficient in Ctla-4. Science.

[32] Klocke K, Sakaguchi S, Holmdahl R, Wing K (2016). Induction of autoimmune disease by deletion of CTLA-4 in mice in adulthood. Proc Natl Acad Sci USA.

[33] Ruppert V, Meyer T, Struwe C, Petersen J, Perrot A, Posch MG, Ozcelik C, Richter A, Maisch B, Pankuweit S (2010). German Heart Failure N. Evidence for CTLA4 as a susceptibility gene for dilated cardiomyopathy. Eur J Hum Genet.

[34] Martini E, Kunderfranco P, Peano C, Carullo P, Cremonesi M, Schorn T, Carriero R, Termanini A, Colombo FS, Jachetti E, Panico C, Faggian G, Fumero A, Torracca L, Molgora M, Cibella J, Pagiatakis C, Brummelman J, Alvisi G, Mazza EMC, Colombo MP, Lugli E, Condorelli G, Kallikourdis M (2019). Single-cell sequencing of mouse heart immune infiltrate in pressure overload-driven heart failure reveals extent of immune activation. Circulation.

[35] Delgobo M, Weiß E, Ashour D, Richter L, Popiolkowski L, Arampatzi P, Stangl V, Arias-Loza P, Mariotti-Ferrandiz E, Rainer PP, Saliba AE, Ludewig B, Hofmann U, Frantz S, Campos RG (2023). Myocardial milieu favors local differentiation of regulatory T cells. Circ Res.

[36] Michel L, Helfrich I, Hendgen-Cotta UB, Mincu RI, Korste S, Mrotzek SM, Spomer A, Odersky A, Rischpler C, Herrmann K, Umutlu L, Coman C, Ahrends R, Sickmann A, Löffek S, Livingstone E, Ugurel S, Zimmer L, Gunzer M, Schadendorf D, Totzeck M, Rassaf T (2022). Targeting early stages of cardiotoxicity from anti-PD1 immune checkpoint inhibitor therapy. Eur Heart J.

[37] Varricchi G, Galdiero MR, Tocchetti CG (2022). Novel actors on the stage of cardiac dysfunction induced by anti-PD1 oncological treatments. Eur Heart J.

[38] Michel L, Totzeck M, Rassaf T (2021). Cardiac dysfunction from cancer and cancer therapy: new pathways for the prevention of late cardiotoxicity. Basic Res Cardiol.

[39] Coman C, Solari FA, Hentschel A, Sickmann A, Zahedi RP, Ahrends R (2016). Simultaneous metabolite, protein, lipid extraction (SIMPLEX): a combinatorial multimolecular omics approach for systems biology. Mol Cell Proteomics.

[40] Xia W, Zou C, Chen H, Xie C, Hou M (2020). Immune checkpoint inhibitor induces cardiac injury through polarizing macrophages via modulating microRNA-34a/Kruppel-like factor 4 signaling. Cell Death Dis.

[41] Du S, Zhou L, Alexander GS, Park K, Yang L, Wang N, Zaorsky NG, Ma X, Wang Y, Dicker AP, Lu B (2018). PD-1 modulates radiation-induced cardiac toxicity through cytotoxic T lymphocytes. J Thorac Oncol.

[42] Palaskas NL, Segura A, Lelenwa L, Siddiqui BA, Subudhi SK, Lopez-Mattei J, Durand JB, Deswal A, Zhao B, Maximilian Buja L, Iliescu C (2021). Immune checkpoint inhibitor myocarditis: elucidating the spectrum of disease through endomyocardial biopsy. Eur J Heart Fail.

[43] Finke D, Heckmann MB, Salatzki J, Riffel J, Herpel E, Heinzerling LM, Meder B, Völkers M, Müller OJ, Frey N, Katus HA, Leuschner F, Kaya Z, Lehmann LH. Comparative transcriptomics of immune checkpoint inhibitor myocarditis identifies guanylate binding protein 5 and 6 dysregulation. Cancers (Basel). 2021;13. 10.3390/cancers13102498 PMC8161064 34065419

[44] Swanson KV, Deng M, Ting JP. The NLRP3 inflammasome: molecular activation and regulation to therapeutics. Nat Rev Immunol. 2019;19(8):477–489. 10.1038/s41577-019-0165-0. 10.1038/s41577-019-0165-0 PMC7807242 31036962

[45] Michel L, Korste S, Spomer A, Hendgen-Cotta UB, Rassaf T, Totzeck M. PD1 deficiency modifies cardiac immunity during baseline conditions and in reperfused acute myocardial infarction. Int J Mol Sci. 2022;23. 10.3390/ijms23147533 PMC9321105 35886878

[46] Ferdinandy P, Andreadou I, Baxter GF, Bøtker HE, Davidson SM, Dobrev D, Gersh BJ, Heusch G, Lecour S, Ruiz-Meana M, Zuurbier CJ, Hausenloy DJ, Schulz R. Interaction of cardiovascular nonmodifiable risk factors, comorbidities and comedications with ischemia/reperfusion injury and cardioprotection by pharmacological treatments and ischemic conditioning. Pharmacol Rev. 2023;75(1):159–216. 10.1124/pharmrev.121.000348 10.1124/pharmrev.121.000348 PMC9832381 36753049

[47] Ferdinandy P, Baczkó I, Bencsik P, Giricz Z, Görbe A, Pacher P, Varga ZV, Varró A, Schulz R (2019). Definition of hidden drug cardiotoxicity: paradigm change in cardiac safety testing and its clinical implications. Eur Heart J.

[48] Reyes-García J, Flores-Soto E, Solís-Chagoyán H, Sommer B, Díaz-Hernández V, García-Hernández LM, Montaño LM (2016). Tumor necrosis factor alpha inhibits L-type Ca(2+) channels in sensitized guinea pig airway smooth muscle through ERK 1/2 pathway. Mediators Inflamm.

[49] Perez-Ruiz E, Minute L, Otano I, Alvarez M, Ochoa MC, Belsue V, de Andrea C, Rodriguez-Ruiz ME, Perez-Gracia JL, Marquez-Rodas I, Llacer C, Alvarez M, de Luque V, Molina C, Teijeira A, Berraondo P, Melero I (2019). Prophylactic TNF blockade uncouples efficacy and toxicity in dual CTLA-4 and PD-1 immunotherapy. Nature.

[50] Schumacher SM, Naga Prasad SV (2018). Tumor necrosis factor-α in heart failure: an updated review. Curr Cardiol Rep.

[51] Wei SC, Meijers WC, Axelrod ML, Anang NAS, Screever EM, Wescott EC, Johnson DB, Whitley E, Lehmann L, Courand PY, Mancuso JJ, Himmel LE, Lebrun-Vignes B, Wleklinski MJ, Knollmann BC, Srinivasan J, Li Y, Atolagbe OT, Rao X, Zhao Y, Wang J, Ehrlich LIR, Sharma P, Salem JE, Balko JM, Moslehi JJ, Allison JP (2021). A genetic mouse model recapitulates immune checkpoint inhibitor-associated myocarditis and supports a mechanism-based therapeutic intervention. Cancer Discov.

[52] Axelrod ML, Meijers WC, Screever EM, Qin J, Carroll MG, Sun X, Tannous E, Zhang Y, Sugiura A, Taylor BC, Hanna A, Zhang S, Amancherla K, Tai W, Wright JJ, Wei SC, Opalenik SR, Toren AL, Rathmell JC, Ferrell PB, Phillips EJ, Mallal S, Johnson DB, Allison JP, Moslehi JJ, Balko JM (2022). T cells specific for α-myosin drive immunotherapy-related myocarditis. Nature.

[53] Ridker PM, Everett BM, Thuren T, MacFadyen JG, Chang WH, Ballantyne C, Fonseca F, Nicolau J, Koenig W, Anker SD, Kastelein JJP, Cornel JH, Pais P, Pella D, Genest J, Cifkova R, Lorenzatti A, Forster T, Kobalava Z, Vida-Simiti L, Flather M, Shimokawa H, Ogawa H, Dellborg M, Rossi PRF, Troquay RPT, Libby P, Glynn RJ (2017). Antiinflammatory therapy with canakinumab for atherosclerotic disease. N Engl J Med.

[54] Merz SF, Korste S, Bornemann L, Michel L, Stock P, Squire A, Soun C, Engel DR, Detzer J, Lörchner H, Hermann DM, Kamler M, Klode J, Hendgen-Cotta UB, Rassaf T, Gunzer M, Totzeck M (2019). Contemporaneous 3D characterization of acute and chronic myocardial I/R injury and response. Nat Commun.

[55] Drobni ZD, Alvi RM, Taron J, Zafar A, Murphy SP, Rambarat PK, Mosarla RC, Lee C, Zlotoff DA, Raghu VK, Hartmann SE, Gilman HK, Gong J, Zubiri L, Sullivan RJ, Reynolds KL, Mayrhofer T, Zhang L, Hoffmann U, Neilan TG (2020). Association between immune checkpoint inhibitors with cardiovascular events and atherosclerotic plaque. Circulation.

[56] Suero-Abreu GA, Zanni MV, Neilan TG (2022). Atherosclerosis with immune checkpoint inhibitor therapy: evidence, diagnosis, and management: JACC: cardiooncology state-of-the-art review. JACC CardioOncol.

[57] Hofmann U, Frantz S (2015). Role of lymphocytes in myocardial injury, healing, and remodeling after myocardial infarction. Circ Res.

[58] Baban B, Liu JY, Qin X, Weintraub NL, Mozaffari MS (2015). Upregulation of programmed death-1 and its ligand in cardiac injury models: interaction with GADD153. PLoS ONE.

